# Assessing the Content Quality of Online Parental Resources about Newborn Metabolic Disease Screening: A Content Analysis

**DOI:** 10.3390/ijns8040063

**Published:** 2022-11-30

**Authors:** Olivia M. Y. Ngan, Wing Ki Wong, Janice Ching Tam, Chi Kong Li

**Affiliations:** 1 Medical Ethics and Humanities Unit, School of Clinical Medicine, LKS Faculty of Medicine, The University of Hong Kong, Hong Kong, China; 2 Faculty of Medicine, The Chinese University of Hong Kong, Hong Kong, China; 3 Department of Paediatrics, Faculty of Medicine, The Chinese University of Hong Kong, Hong Kong, China; 4 Hong Kong Hub of Paediatric Excellence, The Chinese University of Hong Kong, Hong Kong, China

**Keywords:** inherited metabolic diseases, expanded newborn screening, dried blood spot test, urine test, direct-to-consumer, internet, Hong Kong

## Abstract

Parents increasingly utilise the internet to obtain information on health practices, but the quality of online information about screening for inherited metabolic diseases (IMD) needs to be improved. A content analysis examined how IMD blood and urine tests were described online in local healthcare sectors between May and June 2021. Among the nine resources, four were blood test providers and five were urine test providers. All mentioned the test benefits and procedures. Other information, such as false-positive/negative or risk of pain, was infrequently mentioned. The descriptions of urine tests are advertised as outperforming blood tests and can be purchased from commercial laboratory sites without medical guidance. Two urine test providers claimed no false results were reported. A few commercial advertisements highlighted the simplicity of the urine test and potentially overstated the invasiveness of the blood test. We found that some advertisements described IMD as “silent killers” and emphasised the advantage of getting “reassurance” in controlling the child’s developmental health and well-being. To better protect the parents, or broadly, the public interest, regulatory and oversight measures on the urine tests should be implemented to promote the proper use of genetic tests. Without timely regulation and oversight, the incorrect descriptions might create a public misconception about utilising these commercial laboratory tests to inform health decisions.

## 1. Introduction

Inherited metabolic diseases (IMD) are a group of genetic disorders arising from the inherent deficiency of a certain enzyme or cofactor that impairs normal metabolism. Accumulation of excessive toxic substances or deficiency of essential metabolites in the body may damage vital organs, posing a significant risk of morbidity and mortality to patients. A small proportion of IMD can be diagnosed and treated early with diet therapy or pharmacotherapy and may benefit significantly from disease monitoring. As initial symptoms for treatable conditions, e.g., vomiting and characteristic odours [1], are often non-specific and non-exclusive, IMD conditions are likely to be under-detected before symptomatic manifestation. Delayed detection and diagnosis can result in poor health prognosis, severe medical conditions, physical or mental developmental problems, and even death, posing a heavy toll on individuals and the health care system.

Most developed countries have placed great emphasis on extensive IMD screening programmes for treatable conditions to prevent significant developmental delay or severe morbidity and mortality in individuals with treatable IMD conditions. The advancement of tandem mass spectrometry (MS/MS) has led to the rapid development of expanded newborn IMD screening programmes [2], which can efficiently identify infants at increased risk for the conditions in question, enabling specific diagnostic testing and early treatment if warranted to allow for the best possible outcome. The heel-prick blood test (herein blood test) is the most frequently deployed method, examining the subject’s blood sample collected on a filter paper card. The number of disorders screened varies by country, depending on funding, regulations, techniques used, and disease prevalence in the population [3]. Although there are no universal criteria for conditions to be included in the screenings, there is consensus around Wilson and Jungner’s classic screening criteria [4,5] that the screened diseases should have (i) a well-defined natural history, (ii) a high disease prevalence in the general population, (iii) increased mortality and morbidity rates when left untreated, and (iv) effective medical treatment and management plans, such that the results of the screening are meaningful and conducive to improving the health of affected individuals. In the context of newborn screening, disease prevalence is not one of the criteria. Screening a rare disease in the absence of an accepted treatment may be appropriate when it will provide a net benefit to the child or the family [6].

Parental use of the internet to seek health information related to screening is frequently observed during pregnancy [7] and newborn screening processes [8,9,10]. Araia and Potter [11] assessed the content quality of online North American websites on newborn screening programmes and reported an imbalanced emphasis on benefits rather than potential harms. The majority of materials mentioned the purpose and benefits of screening, descriptions of the screening process, and the conditions to be tested. Few sources included information on the possibility of receiving a false-positive or false-negative result, the need for diagnostic follow-up, or the storage or disposal policy of the collected blood spot sample. Although parents often go online and seek information, it is uncertain how they process the online information that determines their health behaviour, such as test uptake or refusal. Given that the internet is one of the most popular sources for medical information among parents, the accuracy and comprehensiveness of the advertised IMD tests play an essential role in parents’ decision-making, their understanding of the test results, and, eventually, the health of the next generation.

In Hong Kong, IMD are common among recognised rare diseases with an incidence rate of 1 in 1376 [12]. In response to public health concerns, in 2015, government-subsidised hospitals instituted a territory-wide newborn metabolic diseases screening programme. Participation in the programme is completely voluntary. Testing, counselling, and follow-up services are centralised at Hong Kong Children’s Hospital. Prior to the launch of the universal screening programme, only newborn cord blood test screening for glucose-6-phosphate dehydrogenase (G6PD) deficiency and congenital hypothyroidism was offered to all newborns free of charge at birth. IMD screening was available only as a pay-per-service test performed in the private healthcare sector. Patients diagnosed with IMD require care from a multidisciplinary team with medical, nursing, and dietetic specialities. Alternatively, IMD screening using blood tests can be done in private clinics and hospitals. The IMD screening using urine tests can be purchased, primarily via the internet, as a self-expensed test. Urine tests were widely used in the early years of the public health programme to screen for certain conditions such as phenylketonuria, by detecting phenyl ketones in the urine. However, they were later replaced by blood tests for higher sensitivity [13,14].

The study objectives are to (1) map the local adoption of IMD screening using blood and urine tests in local public and private sectors, including public hospitals, private hospitals, a university-affiliated private clinic, and commercial laboratories; and (2) evaluate the comprehensiveness of the corresponding published resources. 

## 2. Materials and Methods

### 2.1. Study Design

Between May and June 2021, multiple strategies were adopted to systematically search for, retrieve, and identify expanded newborn metabolic screening tests at local public and private healthcare sectors, including hospitals, clinics, and commercial genetic laboratories. 

After mapping out the suppliers, a cross-sectional content analysis was conducted to examine how the newborn metabolic screening blood and urine tests run by different providers were described in their respective online educational materials. Content analysis is an iterative process of capturing an objective, systematic, and quantitative description of the manifest content of the communication [15,16]. As a research method, it provides a systematic and objective framework to analyse cross-sectional information in all types of documents. It is widely used in various specialities, including antenatal genetic tests [17] and cancer medicine [18]. This study included information pamphlets and websites written in Chinese and/or English, the two official languages of Hong Kong. Given the dynamic nature of online content, screenshots of the websites used for analysis were saved as PDF files and labelled with the date of retrieval. This study involves secondary data analysis and therefore does not require ethical clearance.

### 2.2. Content Abstraction—Data Coding

Table 1 is a standardised data collection instrument codebook drawn from the key messages recommended by the American Academy of Pediatrics Newborn Screening Task Force [19] and the literature [20]. In addition to qualitative coding, the fundamental messages were quantified.

## 3. Results

### 3.1. Information on the Programme

Table 2 describes the current provision of expanded newborn metabolic screening in Hong Kong. Below that is information on the programme in the respective sectors.

#### 3.1.1. Public Sectors

The local Hospital Authority offers a free universal screening programme for IMD to all newborns delivered at public hospitals. The programme covers 26 IMD conditions, including nine organic acid disorders, eight amino acid disorders, and six fatty acid oxidation disorders, and three other conditions. These conditions are selected in concordance with international guidelines and the local context. The information pamphlet for analysis was gathered from the Department of Health website (Document number: NBSIMD/1-60-2/04).

#### 3.1.2. Private Sectors

For those newborns delivered at non-public hospitals, parents need to conduct the test out-of-pocket at private service sectors, including commercial genetic laboratories, university-affiliated hospitals, or private hospitals.

##### Commercial Laboratories

To identify the commercial laboratories offering IMD screening, we conducted systematic searches using two internet search engines—Google Hong Kong and Yahoo—to avoid bias associated with one specific engine. Keywords for internet searches of commercial genetic laboratories were derived from the terminology in the clinical guidelines published in the government documents and the Hospital Authority. The following items used to retrieve relevant articles were designed to include locally appropriate context: (1) newborn screening, (2) Newborn Metabolic Screening Programme, (3) metabolic disorder/disease, or (4) inborn errors of metabolism.

In the end, seven commercial laboratory websites were identified from the online search. Of these, two laboratories offered blood test screening for 48 conditions, and four provided urine test screening for up to 158 conditions. The cost ranged from HKD 1180 to HKD 2900 depending on the laboratory location (local or overseas) and the number of screened conditions.

##### University-Affiliated Clinic

Expanded newborn metabolic screening using blood tests was available as a private service in a university-affiliated clinic starting in July 2013 [21]. It uses a comprehensive model, including pre-test education and counselling, consent-taking procedures, heel-prick blood test, post-test counselling, and treatment follow-up in case of any abnormalities. This service is available to private obstetric or paediatric referrals. Downloadable brochures are available on the university’s website. The programme covers 31 IMD conditions, including 12 organic acid disorders, 9 amino acid disorders, and 10 fatty acid oxidation disorders, alongside congenital adrenal hyperplasia and cystic fibrosis.

##### Private Hospitals

**Figure 1:**
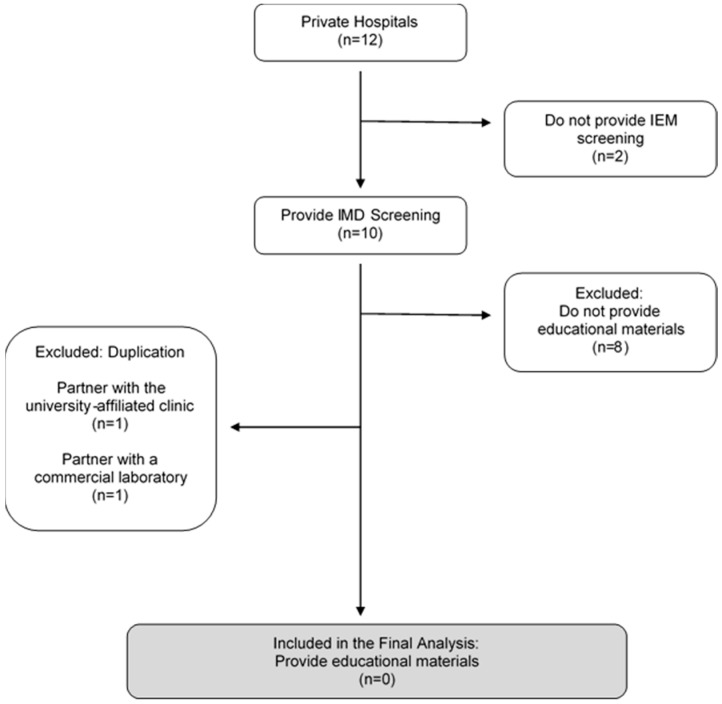
Sampling Pathway for Private Hospitals.

Figure 1 describes the sampling pathway amongst the twelve private hospitals, including ten maternity and two non-maternity hospitals. The team gathered information on the hospital websites. Then, the team wrote an enquiry email and phoned the hotline to collect data from hospitals about information not provided publicly on the internet. A sample of the enquiry email is as follows: (1) Does the hospital provide tests for inborn errors of metabolism? (2) Is it a urine or blood test? (3) How many conditions can be tested? What are the conditions tested? (4) Is the screening limited to inpatient services only?

The two non-maternity hospitals did not provide the test and were excluded. 

Amongst the ten maternity hospitals, only two provided separate pamphlets on IMD blood tests on their hospital website. One of these outsourced the service to the partnering university-affiliated clinic, and another hospital partnered with a commercial genetic laboratory, which was included in the commercial genetic laboratory section. Both are therefore excluded from the content analysis due to duplication.

The team wrote an enquiry email to the remaining eight hospitals that did not post information online. All provided IMD blood test screening for between 26 and 49 conditions. One refused to disclose the number of conditions offered at the hospital. Although they offered IMD blood tests, they did not provide separate pamphlets detailing the test procedures and were therefore excluded from the analysis.

### 3.2. Information on the Programme

**Figure 2:**
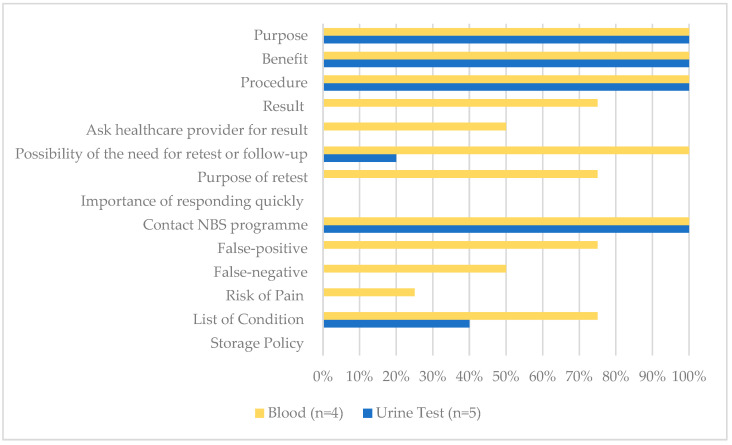
The proportion of all NBS educational materials that included key messages (*n* = 9).

A total of nine resources were identified and retrieved from the following screening programmes: public hospital (*n* = 1), university-affiliated clinic (*n* = 1), and commercial laboratories (*n* = 7). Across all documents, four were blood test providers and five were urine test providers (see Figure 2). None of the materials included all 14 recommended messages, with a mean of 6.67 messages (SD = 0.42). On average, blood test resources mentioned 9.5 messages (SD = 2.06, Range = 7–11), and urine test resources mentioned 4.6 messages (SD = 0.49, Range = 4–5).

#### 3.2.1. Fundamental Message—Blood Tests

All resources correctly categorised blood tests as screening tests to detect the risk of metabolic diseases. They mentioned that the primary purpose of the test is to detect health problems before the apparent symptoms (*n* = 4, 100%). In addition, all noted that the featured benefits of blood tests were early detection and early treatment (*n* = 4, 100%). 

All resources detailed the test procedure of the newborn IMD screening. The blood test must be performed in clinics or hospitals and needs to be done in a short window of time. Two sources offering blood tests suggested that it be performed within 24 to 72 h after birth (50%), and another two (50%) suggested after 24 h and up to the 7th day of life. The average turnaround time ranged from 3 to 7 days. Three (75%) detailed what conditions are covered in the programme. One (25%) stated the need for written consent before the test.

Regarding the test limitations, some mentioned the possibility of receiving a false-positive (*n* = 3, 75%) or false-negative (*n* = 2, 50%) result or identified a risk of pain and/or infection from the procedure (*n* = 1, 25%). One mentioned the possibility of identifying incidental findings (*n* = 1, 25%). None identified how the blood spots would be stored and/or used for other purposes.

Three (75%) mentioned how the result would be presented and interpreted in the categories of “normal”, “positive or abnormal”, and “uncertain or inconclusive”. Two (50%) indicated the involvement of healthcare providers in returning test results. All identified the possibility of a need for re-testing, its purpose and the importance of re-test or follow-up, and included the contact information for the newborn screening programme (*n* = 4, 100%).

#### 3.2.2. Fundamental Message—Urine Tests

Of the five urine test providers, two sources presented a urine test to be both a screening and diagnostic test (40%) concurrently.

Like blood tests, all urine tests (100%) cited early detection and early treatment as the major benefits. Ease of use—in that collecting urine samples is easier than a blood test and this method offered stay-at-home convenience—was also cited as one of the benefits (*n* = 5, 100%). Convenience refers to the accessibility of the test without paying a visit to the clinic with the newborn. Parents can register for the service and obtain the urine collection kit remotely by mail. After collecting the newborn’s urine sample using the provided kit, a courier service is arranged for sample collection. None mentioned the involvement of healthcare providers. 

All claimed to outperform blood tests for several reasons: (1) urine tests involve a non-invasive procedure for collecting a urine sample and thus pose no risk or harm to the newborn; (2) urine tests detect more metabolic conditions than blood tests; (3) urine test resources claim a higher sensitivity, specificity, and flexibility in the detection period, and (4) longer detection period. Urine tests offered a more extended detection period and claimed that they could be performed as early as 48 h after birth and up to 6 months (*n* = 1, 20%), 48 h after birth and up to 14 years old (*n* = 2, 40%), or 3–5 days after delivery and up to 14 years old (*n* = 2, 40%). The average turnaround time ranged from three days to two weeks. Two (40%) provided the list of conditions, and one (20%) mentioned the possibility of re-testing or follow-up.

One (20%) claimed to yield a sensitivity rate of over 99.98%. Two (40%) claimed no false results were reported. No source mentioned how the test would be presented or interpreted or mentioned the need for diagnostic testing to confirm a positive result. The possibility of non-detection or uncertain results was not mentioned across all urine test resources.

## 4. Discussion

This study found that expanded newborn metabolic screening has been disseminated across various healthcare sectors at parents’ disposal with or without professional healthcare assistance. Echoing a North American study [11], our findings showed that most sources were inclined to focus on the benefits and were less likely to highlight the risks or test limitations. For instance, the urine test providers rarely mentioned false-positive or false-negative results or the necessity of a follow-up diagnosis. Other unaddressed essential information included the risk of pain and incidental findings, which, though uncommon, demand attention. As opposed to the claim of having virtually perfect accuracy, screening tests always carry the risk of false results. In a local 18-month prospective study, two mothers were incidentally picked up with IMD of carnitine uptake deficiency (CUD) and classic phenylketonuria [12]. False-negative cases were also reported [20,21]. The complexity of genetic information from IMD screening accentuates the pivotal role of healthcare professionals in pre-test and post-test counselling to explain the meanings of different results. 

We observed some variabilities and incorrect descriptions in introducing the urine tests, both within and across the resources. A few highlighted the simplicity of the urine test and potentially overstated the invasiveness of the blood test. Urine tests also claimed to screen for four times as many conditions as blood tests (150+ and 25+ conditions; see Appendix A and Appendix B). The majority of the screenable conditions are outside of the core panel recommended by the American College of Medical Genetics [22]. Some conditions advertised in the urine test may have been recommended to be identified using the more sensitive dried blood spot card over urine (e.g., phenylketonuria) [13]. Some conditions detected may turn out to be benign, with symptoms often ameliorating and disappearing naturally during follow-up (e.g., Short-Chain Acyl-CoA Dehydrogenase Deficiency) [23]. Some commercial labs claim that urine can be used to perform protein or glucose screening and screen for rare non-childhood conditions such as Meigs syndrome and gestational diabetes mellitus.

Of additional concern, our findings reported that sources incorrectly described the urine test as diagnostic in nature for some diseases that are more sensitive to a blood test (e.g., pyridoxine-5′-phosphate oxidase (PNPO) Deficiency). It is vital to note that the heel prick blood test using MS/MS is proven to be a more efficient and cost-effective technique [24]. The blood test is the standardised method adopted by most, if not all, national screening programmes, including in Hong Kong. The urine test is a diagnostic test for a limited scope of IMD only and not a replacement for conventional blood tests. Most urine-based IMD screenings remained in the research phase, and some were reported to be not sensitive enough for screening [25]. More evidence must be accumulated before these tests are marketed commercially. The healthcare community came to a consensus that blood-based IMD by MS/MS is a good screening test and may require further diagnostic tests. Local tertiary hospital protocols and guidelines listed specific two-tiered approaches and strategic steps to best eliminate inaccurate reporting of results. Appropriate follow-up and professional referral mechanisms should be made available. Further tests as confirmation should always be considered when the first-line tests indicate an increased risk of an IMD. On the opposite end, some commercial laboratory genetic tests that claim to be all-in-one screening and diagnostic tools can thus create a misconception in parents and delay their seeking medical advice.

Hong Kong Chinese parents always want the best care to reassure them about their child’s health. About three-fifths of pregnancies were delivered in the public sector [26], implying that two-fifths of newborn mothers delivered in the private sectors, not benefitting from free-of-charge IMD screening. The latter group are more likely under the influence of commercial resources to take the IMD test. These urine test services are advertised predominantly on the internet and available online at parents’ own choice and expense without medical guidance. The popular notion of “the more you know about the child, the better” is commonly reported among parents for better planning regarding the child’s development [27]. Without a comprehensive understanding of what conditions are necessary to be screened, parents may be under the impression that a greater number of screenable conditions would be the best option. 

Zayts and Luo [28] studied the language used by commercial laboratories in Hong Kong. They found that online materials tend to use sentimental terms or make inflated claims about the value of genetic tests to pursue a marketing agenda. In concordance with the results of Zayts and Luo, we found that some advertisements described IMD as “silent killers” and emphasised the advantage of getting “reassurance” in controlling the child’s developmental health and well-being. The use of storytelling and linguistic foregrounding is prevalent and potentially biased in promoting health, making targeted advertisement audiences relatable to these shared parental responsibilities, emotions, and narratives. It is unsurprising that the public may have been misled easily and overestimated the utility of the commercial laboratory test. Making an informed choice under the influence of biased information and in the absence of health care providers is also questionable, especially considering the Hong Kong Chinese population has attained a low genomic literacy [29]. Genetic counsellors’ views towards any commercial laboratory tests were nuanced. They asserted that these commercial tests should be provided responsibly with appropriate access to information, an informed consent process, and professional advice [30]. To better protect the parents, or broadly, the public interest, the Steering Committee on Genomic Medicine issued strategic plans for developing genomic medicine. One of the scopes is to recommend pre-testing consultation with qualified healthcare professionals [31]. In Hong Kong, the development of genetic counselling as a licensing profession is lagging behind compared with other developed regions and countries [32]. Recognising the limited capacities in clinical genetics and bioinformatics, the Steering Committee recommended that academic institutes offer relevant internationally certified programmes to meet the burgeoning service demand [31]. Another indicative action is promoting the proper use of genetic and genomic tests. Implementing additional regulatory measures on commercial tests from a top-down approach is crucial yet complex, varying by country. For example, France and Germany ban commercial genetic tests and mandate the involvement of healthcare professionals in any genetic examination [33]. Like many regions and countries, Hong Kong does not provide legislation governing commercial laboratory tests. These tests are recognised as goods under the law unless false trade descriptions and inaccurate, misleading or incomplete information regarding the goods provided are evident. Without timely regulation and oversight, the incorrect descriptions might create a public misconception about utilising these commercial laboratory tests to inform health decisions. 

## 5. Study Limitations

Our evaluation of IMD resources should be interpreted in the context of limitations. First, our sampling method enabled us to evaluate the status and content of online sources pertinent to IMD in the Hong Kong Chinese population. Second, we offer insights based on a limited analysis time (between May and June 2021). Aware of the evolving technology, in that the content will vary over time, we controlled for temporal factors by taking screenshots of the content sources. Despite these limitations, this study fills in the literature gap as the first analysis of internet resources regarding newborn screening for IMD.

## Figures and Tables

**Table 1:** Fundamental Messages Recommended by Literature.

Theme	Definition
Purpose	The test detects health problems that would not or might not be apparent without testing
Benefits	The test may prevent serious health problems
Sampling Procedure	How testing is performed
Results	How will you receive the results
Healthcare Provider Involvement	You may ask your healthcare providers for the results
Purpose of Re-test	There is a possibility of re-testing or follow-up
Importance of Re-test	The importance of responding quickly to a request for follow-up testing
Contact	How to contact the test programme
False-positive	Possibility of receiving a false-positive result
False-negative	Possibility of receiving a false-negative result
Risks	Risk of pain or infection
List of Conditions	A list of the conditions screened
Storage Policy	Information about policies and practices related to the storage and use of the bio-sample

**Table 2:** Summary of Newborn IMD Screening by Sector.

					Number of Conditions				
Code	Sectors	Include in Analysis	Type of Test	Online Sources	Total	Organic Acid	Amino Acid	Fatty Acid	Other
Public Sectors									
G1	Public Hospital	Yes	Blood (MS/MS)	Yes	26	8	9	6	3
Private Sectors									
L1	Commercial Laboratory	Yes	Blood (MS/MS)	Yes	48	12	21	15	0
L2	Commercial Laboratory	Yes	Blood (MS/MS)	Yes	48	12	21	15	0
L3	Commercial Laboratory	Yes	Urine (GC/MS)	Yes	106 ^#^	-	-	-	-
L4	Commercial Laboratory	Yes	Urine (GC/MS)	Yes	106	59		6	41
L5	Commercial Laboratory	Yes	Urine (GC/MS)	Yes	106	59		6	41
L6	Commercial Laboratory	Yes	Urine (GC/MS)	Yes	158	-	-	-	-
L7	Commercial Laboratory	Yes	Urine (GC/MS)	Yes	158	-	-	-	-
U1	University-affiliated Clinic	Yes	Blood (MS/MS)	Yes	33	12	9	10	2
P1	Maternity Hospital	No ^^^	Blood (MS/MS)	No	- ^#^	-	-	-	-
P2	Maternity Hospital	No ^^^	Blood (MS/MS)	No	26	8	9	6	3
P3	Maternity Hospital	No ^^^	Blood (MS/MS)	No	33	12	9	10	2
P4	Maternity Hospital	No ^^^	Blood (MS/MS)	No	33	12	9	10	2
P5	Maternity Hospital	No ^^^	Blood (MS/MS)	No	33	12	9	10	2
P6	Maternity Hospital	No ^^^	Blood (MS/MS)	No	33	12	9	10	2
P7	Maternity Hospital	No ^^^	Blood (MS/MS)	Yes	33	12	9	10	2
P8	Maternity Hospital	No ^^^	Blood (MS/MS)	No	31	11	10	10	0
P9	Maternity Hospital	No ^^^	Blood (MS/MS)	Yes	48	12	21	15	0
P10	Maternity Hospital	No ^^^	Blood (MS/MS)	No	49	11	17	15	6

^^^ Excluded from the analysis as the unit outsourced IMD using commercial laboratory or university-affiliated clinic. ^#^ Refused to disclose the number of conditions or unavailable online

## Data Availability

The data that support the findings of this study are available on reasonable request from the corresponding author.

## Appendix A

**Table A1:** A comparison of IMD conditions screened by blood test providers with the American College of Medical Genetics and Genomics (ACMG) Screening Panel.

#	Inborn Errors of Metabolism	Code	ACMG Classification *	L1	L2	U1	G1
**Amino Acid Disorders**							
1	Argininosuccinic Aciduria	ASA	Core	Yes	Yes	Yes	Yes
2	Citrullinaemia Type 1	CITI	Core	Yes	Yes	Yes	Yes
3	Homocystinuria/Homocystinemia	HCU	Core	Yes	Yes	Yes	Yes
4	Maple Syrup Urine Disease	MSUD	Core	Yes	Yes	Yes	Yes
5	Phenylketonuria	PKU	Core	Yes	Yes	Yes	Yes
6	Tyrosinaemia Type 1	TYR I	Core	Yes	Yes	Yes	Yes
7	Citrullinaemia Type 2	CIT II	2	Yes	Yes	Yes	Yes
8	Arginase Deficiency	ARG	2	Yes	Yes	Yes	Yes
9	Defects of Biopterin Cofactor Biosynthesis and Regeneration	BIOPT (BS & REG)	2	-	-	Yes	-
10	Tyrosinaemia II	TYR II	2	Yes	Yes	-	-
11	Tyrosinaemia III	TYR III	2	Yes	Yes	-	-
12	6-Pyru’voyl-Tetrahydropterin Synthase Deficiency	PTPS	-	-	-	-	Yes
13	Carbamyl Phosphate Synthase Deficiency	CPS	-	Yes	Yes	-	-
14	Histidinemia	-	-	Yes	Yes	-	-
15	Hyperammonemia	-	-	-	Yes	-	-
16	Hypermethioninemia Hyperornithinemia Homocitrullinuria Syndrome	HHH	-	Yes	Yes	-	-
17	Hyperornithinemia	-	-	Yes	Yes	-	-
18	Hyperornithinemia with Gyralatrophy	HOGA	-		Yes	-	-
19	Hypervlinemia	-	-	Yes	Yes	-	-
20	Hypermethioninemia	-	-	Yes	-	-	-
21	Hyperphenylalaninemia	-	-	Yes	-	-	-
22	Tetrahydrobiopterin Deficiency	BH4D	-	Yes	Yes	-	-
23	N-Acetyglutamate Synthase Deficiency	NAGS	-	Yes	Yes	-	-
24	Non Ketotic Hyperglycinemia	NKH	-	Yes	Yes	-	-
25	Ornithine Transcarbamylase Deficiency	OTO	-	Yes	Yes	-	-
**Fatty Acid Oxidation Disorders**							
26	Carnitine Update Defect	CUD	Core	Yes	Yes	Yes	Yes
27	Long-Chain 3-Hydroxyl-Acyl-CoA Dehydrogenase Deficiency	LCHAD	Core	Yes	Yes	Yes	-
28	Medium-Chain Acyl-CoA Dehydrogenase Deficiency	MCAD	Core	Yes	Yes	Yes	Yes
29	Trifunctional Protein Deficiency	TFP	Core	Yes	Yes	Yes	-
30	Very Long-Chain Acyl-CoA Dehydrogenase Deficiency	VLCAD	Core	Yes	Yes	Yes	Yes
31	2,4- Dienoyl-CoA Reductase Deficiency	DE RED	2	Yes	Yes	-	-
32	Carnitine Palmitoyltransferase I Deficiency	CPTI	2	Yes	Yes	Yes	-
33	Carnitine Palmitoyltransferase II Deficiency	CPTII	2	Yes	Yes	Yes	Yes
34	Carnitine-Acylcarnitine Translocase Deficiency	CACT	2	Yes	Yes	Yes	Yes
35	Glutaric Acidaemia Type II	GAII	2	Yes	Yes	-	Yes
36	Medium/Short-Chain Hydroxyl-Acyl-CoA Dehydrogenase	M/SCHAD	2	Yes	Yes	Yes	-
37	Medium-Chain Ketoacyl-CoA Thiolase Deficiency	MCKAT	2	Yes	Yes	-	-
38	Short-Chain Acyl-CoA Dehydrogenase Deficiency	SCAD	2	Yes	Yes	Yes	-
39	Ethylmalonic Encephalopathy	EE	-	Yes	Yes	-	-
40	Malonyl-CoA Decarboxylase Deficiency	MCD	-	Yes	Yes	-	-
**Organic Acid Disorders**							
41	3-Hydroxy-3-Methylglutaryl-CoA Lyase Deficiency	HMG	Core	Yes	Yes	Yes	Yes
42	3-Methylcrotonyl-CoA Carboxylase Deficiency	3MCC	Core	Yes	Yes	Yes	-
43	Glutaric Aciduria Type 1	GAI	Core	Yes	Yes	Yes	Yes
44	Isovaleric Aciduria	IVA	Core	Yes	Yes	Yes	Yes
45	Methylmalonic Aciduria	Cbl A,B	Core	?	-	Yes	-
46	Methylmalonic Aciduria	MUT	Core	?	Yes	Yes	Yes
47	Multiple Carboxylase Deficiency	MCD	Core	Yes	Yes	Yes	Yes
48	Propionic Acidaemia	PA	Core	Yes	Yes	Yes	Yes
49	ß-Ketothiolase Deficiency	BKT	Core	Yes	Yes	Yes	Yes
50	3-Methylglutaconic Aciduria Type I	3MGA	2	Yes	Yes	Yes	-
51	Methylmalonic Aciduria	Cbl C,D	2	?	-	Yes	Yes
52	Malonic Aciduria	MAL	2	-	-	Yes	-
53	2-Methyl-3-Hydroxybutyryl-CoA Aciduria	2M3HBA	2	Yes	Yes	-	-
54	2-Methylbutyryl-CoA Dehydrogenase Deficiency	2MBG	2	Yes	Yes	-	-
55	Isobutyryl-CoA Dehydrogenase Deficiency	IBD	2	Yes	Yes	-	-
**Others**							
55	Biotinidase Deficiency	BIOT	Core	-	-	-	Yes
56	Congenital Adrenal Hyperplasia	CAH	Core	-	-	Yes	Yes
57	Cystic Fibrosis	CF	Core	-	-	Yes	-
58	X-linked adrenoleukodystrophy	ALD	Core	-	-	Yes ^^^	-
59	Classic Galactosaemia	GALT	Core	-	-	-	Yes
60	Galactose Epimerase Deficiency	GALE	2	-	-	-	-
61	Galactokinase deficiency	GALK	2	-	-	-	-
62	Severe Combined Immunodeficiency	SCID	-	-	-	Yes ^^^	Yes ^#^
63	Spinal Muscular Atrophy	SMA	-	-	-	Yes ^^^	-

* Core conditions refer to the diseases included in the mandated newborn screening in the United States. 2 conditions refer to the secondary diseases that are part of the differential diagnosis of a core condition and are revealed with the screening technology. They are clinically significant but lack an efficacious treatment or represent incidental findings for which there is potential clinical significance. ? Did not specify the type. ^^^ Added in the screening panel after the analysis period ^#^ The condition is under pilot testing under the public healthcare system after the analysis period.

## Appendix B

**Table A2:** A list of conditions screened by the urine test.

1. 2-Oxoadipic Aciduria 2. 3-Hydroxy 3-Methyl Glutaric Aciduria 3. 3-Hydroxyisobutyryl-CoA Deacylase Deficiency 4. 3-Methylcrotonyl-CoA Carboxylase Deficiency 5. 3-Methylglutaconic Aciduria (Type I) 6. 3-Methylglutaconic Aciduria (Type II) 7. 3-Methylglutaconic Aciduria (Type III) 8. 3-Methylglutaconic Aciduria (Type IV) 9. 3-Methylglutaconic Aciduria (Type V) 10. 5-Oxoprolinuria 11. 5,10-Methylenetetrahydrofolate Reductase (MTHFR) Deficiency 12. ß-Ureidopropionase Deficiency 13. Adenine Phosphoribosyltransferase Deficiency 14. Adenosine Deaminase Deficiency 15. Adenylosuccinate Lyase Deficiency (ASLD) 16. Adult-onset Type II Citrullinemia (CTLN2) 17. Alkaptonuria 18. Aminoadipic Aciduria 19. Argininemia 20. Argininosuccinase Deficiency 21. Argininosuccinate Synthase Deficiency 22. Aspartylglucosaminuria (AGU) 23. Aspirin Poisoning 24. Biotinidase Deficiency 25. Canavan Disease 26. Carbamoyl Phosphate Synthetase Deficiency 27. Carnitine Palmitoyl Synthase Deficiency (CPSD) 28. Carnitine Palmitoyl Synthase I Deficiency (CPSID) 29. Carnitine Palmitoyl Synthase II Deficiency (CPSIID) 30. Carnitine Transport Defect 31. Citrullinemia 32. Cystathioninuria 33. Cystinuria 34. Diabetes Mellitus Type I 35. Diabetes Mellitus Type II 36. Dicarboxylic Aciduria 37. Dihydrolipoyl Dehydrogenase (E3) Deficiency 38. Dihydropyridinase Deficiency 39. Dihydropyrimidine Dehydrogenase Deficiency 40. Endogenous Sucrosuria 41. Ethanolaminosis 42. Ethylene Glycol Poisoning 43. Ethylhydracrylic Aciduria 44. Fanconi Syndrome 45. Formiminoglutamic Aciduria 46. Fructosuria 47. Fructose-1, 6-Diphosphatase Deficiency 48. Fumaric Aciduria 49. Galactosemia I 50. Galactosemia II 51. Galactosemia III 52. Gestational Diabetes Mellitus 53. Gluconeogenesis Disorder 54. Glutaric Aciduria Type I 55. Glutathione Synthetase Deficiency 56. Glyceric Aciduria 57. Glyceroluria 58. Hartnup Syndrome 59. Hawkinsinuria 60. Hepatic Failure 61. Hepatic Tyrosinemia 62. Hereditary Fructose Intolerance 63. Hereditary Xanthinuria 64. Histidinemia 65. Holocarboxylase Synthetase Deficiency 66. Homocystinuria 67. Hemosiderinuria 68. Hydroxyprolinemia 69. Hypernoxaluria Type I 70. Hyper- β -Alaninemia 71. Hyperoxaluria Type Il 72. Hyperdibasicaminoaciduria 73. Hyperglycinuria 74. Hyperleucinemia 75. Hyperleucinuria 76. Hyperlysinemia 77. Hypermethioninemia 78. Hyperornithinemia-hyperammonemia-homocitrullinuria (HHH) syndrome 79. Hyperornithinemia 80. Hyperphenylalaninemia 81. Hyperprolinemia Type I, Prolidase Deficiency 82. Hypersarcosinemia 83. Hypervalinemia 84. Hypervalinemia 85. Hypophosphatasia 86. Hypoxanthine Adenine Phosphoribosyltransferase Deficiency 87. Infantile Refsum Disease 88. Intestinal Bacterial Overgrowth 89. Isovaleric Acidemia 90. Ketoadipic Aciduria 91. Lactic Acidemia 92. Lactose Intolerance 93. Lesch-Nyhan Syndrome 94. Long-chain acyl-CoA Dehydrogenase Deficiency 95. Long-chain 3-hydroxyacyl-CoA Dehydrogenase (LCHAD) Deficiency 96. Lysine Malabsorption 97. Lysinuric Protein Intolerance 98. Malabsorption Syndromes 99. Malonyl-CoA Decarboxylase Deficiency 100. Maple Syrup Urine Disease 101. MCT Oil Fed Dicarboxylic Aciduria 102. Medium Chain Acyl-CoA Dehydrogenase Deficiency 103. Menkes Disease 104. Mercaptolactate-Cysteine Disulfiduria 105. Methylmalonic Acidemia (Cbi D-HC) 106. Methylmalonic Acidemia (Cbi D-MMA/HC) 107. Methylmalonic Acidemia (Cbi E) 108. Methylmalonic Acidemia (Cbi F) 109. Methylmalonic Acidemia (Cbi G) 110. Methylmalonic Acidemia (Cbl A) 111. Methylmalonic Acidemia (Cbl B) 112. Methylmalonic Acidemia (Cbl C) 113. Methylmalonic Acidemia (MUT) 114. Methylmalonic Acidemia (Muto) 115. Methylmalonic Acidemia (Vitamin B 12 Deficiency) 116. Mevalonate Kinase Deficiency 117. Mitochondrial Trifunctional Proscin Deficiency (MTPD) 118. Molybdenum Cofactor Deficiency/Sulfite Oxidase Deficiency 119. Multiple acyl-CoA Dehydrogenase Deficiency 120. N-Acetylglutamate Synthase Deficiency 121. Neonatal Adrenoleukodystrophy 122. Neonatal Intrahepatic Cholestasis (NICCD) 123. Neuroblastoma 124. Normal Fed Medium Chain Triglyceride Formulas Dicarboxylic Aciduria 125. Ornithine Transcarbamylase Deficiency 126. Orotic Aciduria 127. Pentosuria 128. Phenylketonuria 129. Pheochromocytoma 130. Primary Hyperoxaluria 131. Propionic Acidemia 132. Pyridoxamine 5-Phosphate Oxidase Deficiency 133. Pyruvate Carboxylase Deficiency 134. Pyruvate Dehydrogenase Deficiency 135. Renal Dysfunction 136. Renal Glycosuria 137. Saccharopinuria 138. Short Chain Acyl-CoA Dehydrogenase Deficiency 139. Short-Chain 3-Hydroxyacyl-CoA Dehydrogenase Deficiency (SCHAD) 140. Sialic Acid Storage Disease 141. Succinic Semialdehyde Dehydrogenase Deficiency (4-Hydrozybutyric Aciduria) 142. Succinyl-CoA:3-ketoacid CoA Transferase Deficiency 143. Tegretol Poisoning 144. Tetrahydrobiopterin (BH4) Deficiency 145. Transient Galactosemia 146. Transient Neonatal Tyrosinemia 147. Trimethylaminuria 148. Tryptophanuria 149. Tyrosinemia Type 1 150. Tyrosinemia Type III 151. Tyrosinemia Type Il 152. Valproate Toxicity 153. Very Long-Chain Acyl-CoA Dehydrogenase Deficiency (VLCAD) 154. Vitamin B12 Deficiency or Malabsorption 155. Zellweger Syndrome 156. Zellweger-Like Syndrome 157. ß-Ketothiolase (T2) Deficiency 158. ß-Aminoisobutyric Aciduria (BAIB)
